# A correlation between grain boundary character and deformation twin nucleation mechanism in coarse-grained high-Mn austenitic steel

**DOI:** 10.1038/s41598-021-87811-w

**Published:** 2021-04-19

**Authors:** Chang-Yu Hung, Yu Bai, Tomotsugu Shimokawa, Nobuhiro Tsuji, Mitsuhiro Murayama

**Affiliations:** 1 Department of Materials Science and Engineering, Virginia Tech, Blacksburg, VA 24061 USA; 2 Department of Materials Science and Engineering, Kyoto University, Yoshida-honmachi, Sakyo-ku, Kyoto, 606-8501 Japan; 3 Faculty of Mechanical Engineering, Kanazawa University, Kanazawa, Ishikawa 920-1192 Japan; 4 Elements Strategy Initiative for Structural Materials (ESISM), Kyoto University, Yoshida-honmachi, Sakyo-ku, Kyoto, 606-8501 Japan; 5 Institute for Materials Chemistry and Engineering, Kyushu University, Kasuga, Fukuoka 816-8580 Japan

**Keywords:** Materials science, Structural materials, Metals and alloys

## Abstract

In polycrystalline materials, grain boundaries are known to be a critical microstructural component controlling material’s mechanical properties, and their characters such as misorientation and crystallographic boundary planes would also influence the dislocation dynamics. Nevertheless, many of generally used mechanistic models for deformation twin nucleation in fcc metal do not take considerable care of the role of grain boundary characters. Here, we experimentally reveal that deformation twin nucleation occurs at an annealing twin (Σ3{111}) boundary in a high-Mn austenitic steel when dislocation pile-up at Σ3{111} boundary produced a local stress exceeding the twining stress, while no obvious local stress concentration was required at relatively high-energy grain boundaries such as Σ21 or Σ31. A periodic contrast reversal associated with a sequential stacking faults emission from Σ3{111} boundary was observed by in-situ transmission electron microscopy (TEM) deformation experiments, proving the successive layer-by-layer stacking fault emission was the deformation twin nucleation mechanism, different from the previously reported observations in the high-Mn steels. Since this is also true for the observed high Σ-value boundaries in this study, our observation demonstrates the practical importance of taking grain boundary characters into account to understand the deformation twin nucleation mechanism besides well-known factors such as stacking fault energy and grain size.

## Introduction

Deformation by twinning in face centered cubic (fcc) metals has been studied extensively since 1950s, however, besides the stacking fault energy (SFE), the details of governing factors to determine the operative deformation twin nucleation mechanism have yet to be shown. Austenitic high-manganese (Mn) steels with a single fcc matrix phase are one of representative alloy systems for the twinning induced plasticity (TWIP)^1–5^, and it is suitable to study the SFE—deformation behavior correlation due to its ability to tune the SFE by adjusting the alloys’ chemical compositions and its industrial value. Recent in-situ transmission electron microscopy studies^6–8^ have demonstrated that deformation mechanism in high-Mn steels changes according to the SFE of the alloys. Deformation twin nucleation associated with both perfect and Shockley partial dislocations was observed in a low SFE high-Mn alloy (SFE ~ 12 mJ/m^2^), whereas plastic deformation was governed by planer dislocation glide in a high SFE counterpart (SFE ~ 85 mJ/m^2^). It is commonly believed that high-Mn steels are roughly categorized into three groups based on their SFE, i.e., low-SFE (< 20 mJ/m^2^), medium-SFE (20–40 mJ/m^2^) and high-SFE (> 40 mJ/m^2^). The level of SFE changes the deformation twining nucleation by changing dislocation dissociation behavior thus it influences the strain hardening response of high-Mn TWIP steels. On the other hand, the high SFE case indicates that the SFE may not be the sole governing factor when compared these results with the deformation behavior of pure fcc metals such as copper (SFE ~ 70 mJ/m^2^).

Similarly, grain size is known to influence the operative deformation mode of the high-Mn TWIP steels by altering the nucleation site of carriers of plastic deformation, i.e., dislocations, deformation twins, and martensitic transformation^9–12^. The reasons for the deformation mode change are not fully understood, but may be attributed to the nature of grain boundaries and their role of deformation twin nucleation, in addition to the volume confinement effect on the grain-interior dislocation source activity, known as dislocation source hardening^13, 14^.

So far, there are five proposed deformation twin nucleation mechanisms applicable to high-Mn TWIP steels: that are Venables pole mechanism^15^, Fujita-Mori stair-rod cross-slip mechanism^16^, Cohen-Weertman-Frank cross-slip mechanism^17^, Miura-Takamura-Narita primary slip mechanism^18^, and Mahajan-Chin three-layer faults mechanism^19^. They are based on microstructure investigations by transmission electron microscopy (TEM) and commonly indicate (a) a sufficient dislocation density in a grain and/or local stress concentration as essential prerequisites, and (b) an arrangement of highly coordinated Shockley partial dislocations glide on {111} slip planes, are the key features of the deformation twinning process. Meanwhile, none of these mechanisms extensively argued the role of grain boundary, i.e., grain boundary characters. Historically, experimental observations focused on deformation twin nucleation behavior on {111} twin boundaries, because the annealing twin boundaries are most likely sites for deformation twin nucleation based on electron backscattered diffraction (EBSD) analyses^20^. However, a recent work indicates that the deformation twins are nucleated from the vicinity of a grain boundary rather than exactly at the grain boundary based on a series of selected area electron diffraction (SAED) analyses^21^. Generally speaking, grain boundary structure and misorientation affect various physical properties of materials including plasticity^22–30^. The structural units and their sequences within a grain boundary also significantly affect the dislocation nucleation process, thus these factors are expected to be influential to the nucleation site of carriers of plastic deformation as much as the SFE and grain size. A recent computational study indicates that intergranular interactions could influence the local strain distribution and strain transfer near grain boundary^31^, leaving open questions, i.e., whether the grain boundary character such as misorientation and boundary plane structure would regulate (i) the deformation twinning nucleation mechanisms associated with a grain boundary and (ii) dislocation dynamics and the deformation twining precursor structure near/at the grain boundary.

This study aims to clarify the correlation between the grain boundary character and deformation twin nucleation mechanism, and to directly observe the nucleation process of deformation twin precursor in near/at grain boundary region. A coarse-grained Fe-31Mn-3Al-3Si (wt%) high manganese TWIP steel (SFE = 40 mJ/m^2^) was used a model alloy for medium SFE high-Mn steel, the deformation twin nucleation mechanism at a low-energy Σ3{111} and several high sigma-value boundaries were investigated.

## Results

EBSD analyses showed that nearly 40% grain boundaries in this alloy were identified as Σ3{111} (twin) boundary by taking more than 200 grain boundaries into account. The Σ3{111} boundaries were determined as the [111]/60° axis/angle pair, which were basically annealing twin boundary. A histogram in Supplementary Fig. S1 indicates the population of coincidence site lattice (CSL) boundaries having a certain axis/angle pair obtained by EBSD analysis^32^. Besides Σ3{111}, the sum of all other CSL boundaries (Σ5 to Σ49) over the total boundaries was estimated to be 6%. Since Σ3{111} boundary is the dominant type boundary in this alloy and also predominantly discussed in previous studies, its deformation behavior will be carefully examined and compared with that of high-sigma value counterparts.

Symbols used in TEM images are listed in Table 1. Each of symbols indicates the direction of reciprocal lattice g-vector excited to image, streak in electron diffraction pattern, dislocation gliding direction, stacking faults, and dislocations.

**Table 1:** List of symbols used in TEM images.

Symbol	Description
	g-vector direction
	Streak (in electron diffraction pattern)
	Dislocation gliding direction
	Stacking fault
	Dislocation

### Annealing twin (Σ3{111}) boundaries

**Figure 1:**
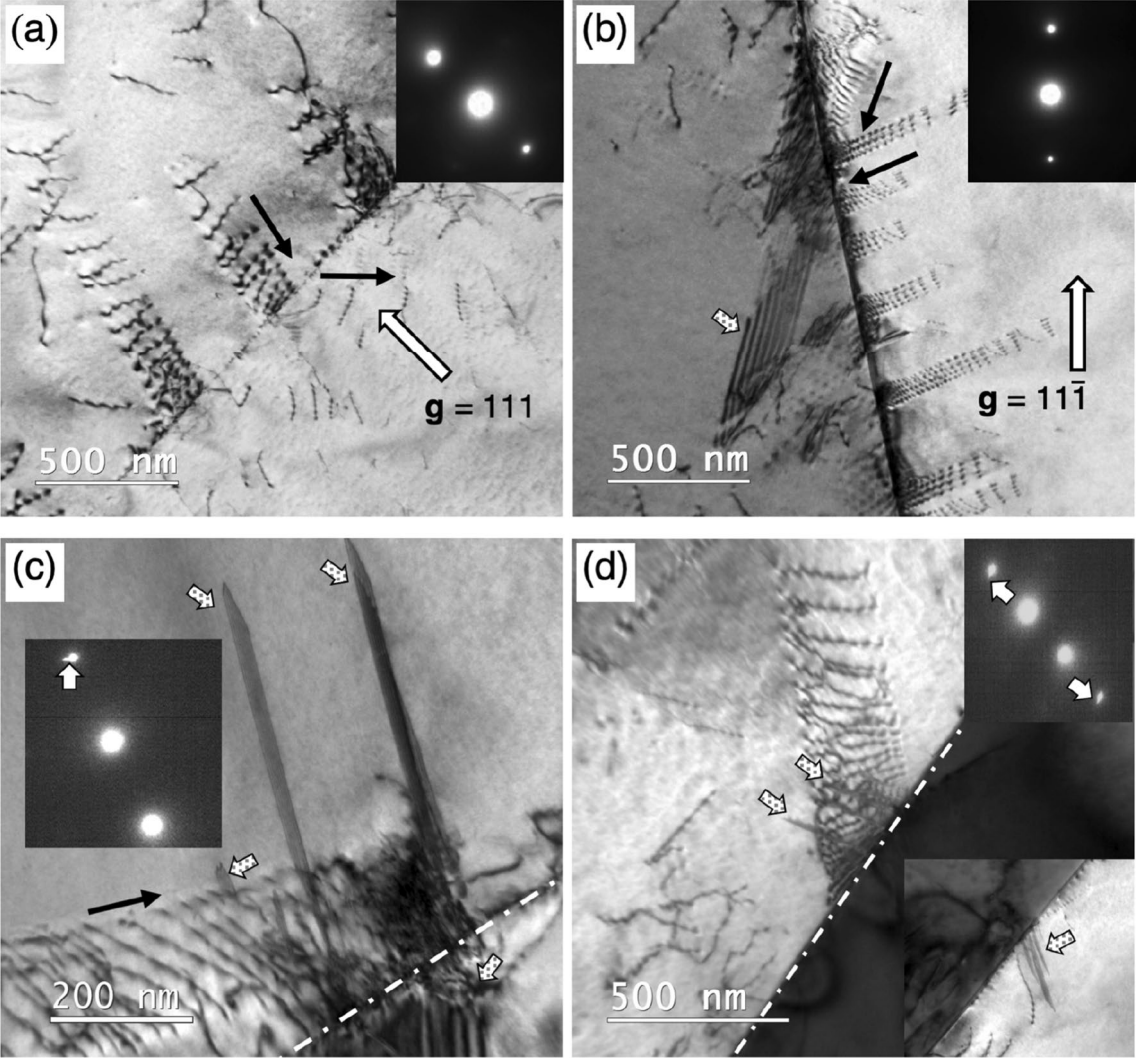
BF TEM micrographs showing the representative microstructure and structural defects in the TWIP steel deformed to engineering strain of 0.01 (**a**,**b**) and 0.02 (**c**,**d**). (**a**) Dislocations gliding and inducing the slip transfer from the starting grain to the twinned grain, taken in a two-beam condition for **g** = ${111}$. (**b**) A stacking fault near the annealing twin boundary (indicated by an arrow filled with dots) and several dislocations in the right-side grain impinged the annealing twin boundary, taken in a two-beam condition for **g** = ${11}\bar{1}$. (**c**,**d**) Dislocations pile-ups and stacking faults nucleation observed near an annealing twin boundary. The emitted stacking faults are indicated by the arrows filled with dots. The corresponding SAED patterns in (**c**,**d**) show a fine streak (white arrows), attributed to the shape factor of a planar fault.

Bright field (BF) TEM images of Σ3{111} (annealing twin) boundaries after deformed to engineering tensile strains of 0.01 and 0.02 were taken at different two-beam diffraction conditions in Fig. 1. Each image shows different grain boundary–dislocation interactions in or near boundaries. Figure 1a, the operative reciprocal lattice vector **g** = 111, illustrates dislocations gliding and inducing the slip transfer from the upper-left starting grain to the lower-right twinned grain, i.e., a continuous slip transfer across the Σ3{111} boundary. In contrast, Fig. 1b, the operative reciprocal lattice vector **g** = ${11}\bar{1}$, shows a stacking fault (indicated by an arrow filled with dots) near a Σ3{111} boundary in the left grain and dislocations impinged on the opposite side of the boundary, i.e., the formation of stacking faults. It should be noted that the dislocations’ gliding direction shown in black arrows were estimated from the changes in their curvature.

The continuous slip transfer across grain boundary could take place when the line of intersection defined by incoming and outgoing slip planes on the grain boundary is colinear and the Burgers vectors of screw dislocations of incoming and outgoing slip systems are identical^33^. On the other hand, the stacking fault nucleation at the site where the non-screw dislocations impinged may take place when non-screw dislocations get incorporated into the grain boundary^34, 35^. It is worth noting that the continuous slip transfer across a Σ3{111} boundary was rarely observed in this study compared with the stacking fault nucleation. This may suggest that dislocations reached and impinged at annealing twin boundary tend to have an edge component rather than pure screw.

At an engineering strain of 0.02, larger number of planer defect like contrasts were observed near grain boundaries. The planer defects in Fig. 1c,d (arrows filled with dots) were clarified to be stacking faults by examining their fringe contrast and weak streaks arising in selected area electron diffraction (SAED) patterns. The formation of stacking faults in this strain level was observed on the both sides of a Σ3{111} grain boundary. These deformation-induced stacking faults are expected to transform into deformation twins through a sequential formation of Shockley partial dislocations on the {111} slip planes if the sample will continuously be deformed as reported by previous studies^36–38^.

**Figure 2:**
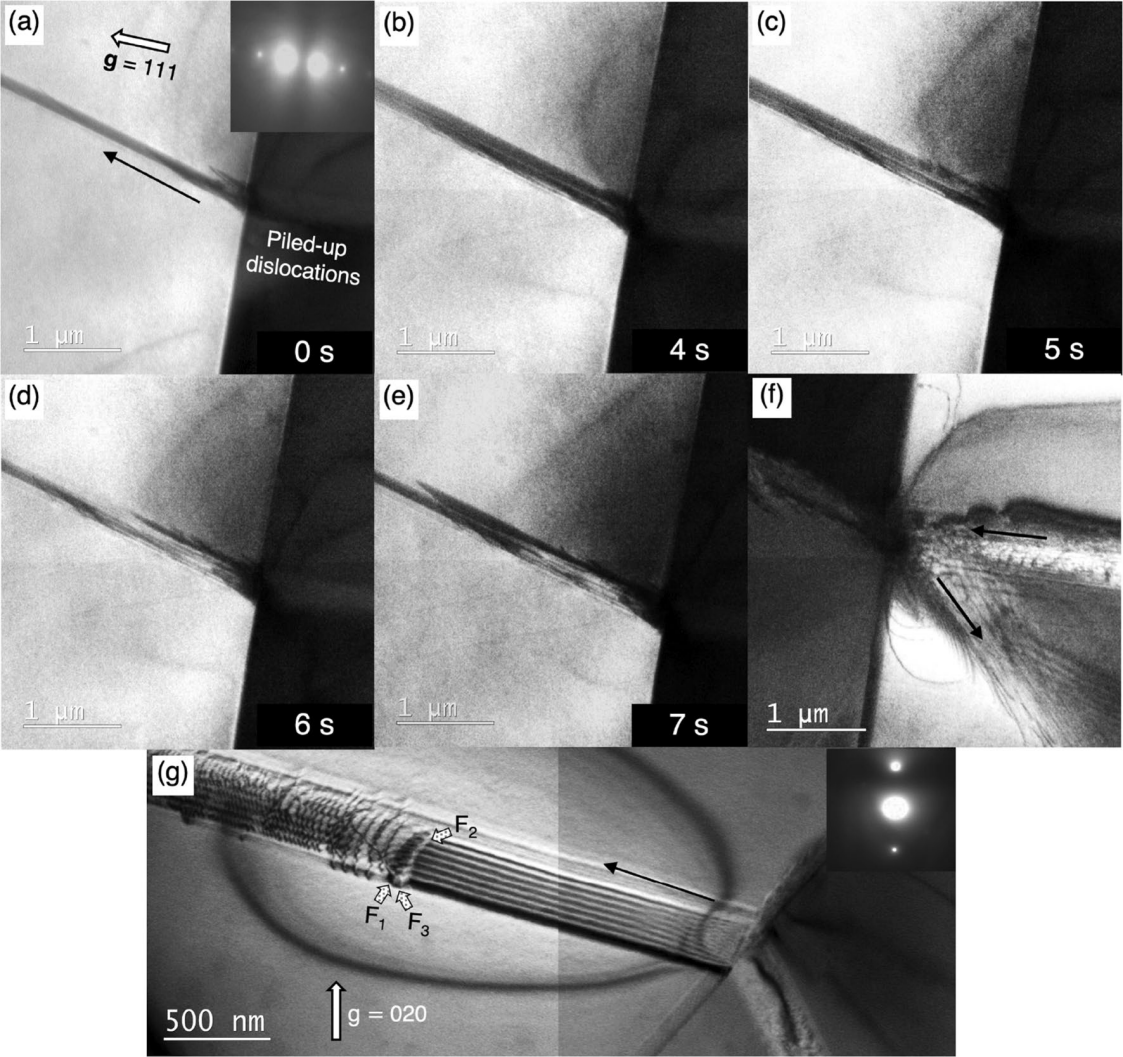
Selected frames of an in-situ deforming TEM test video data (see the original video, Supplementary Video S1 online) showing a near annealing twin boundary region. The video was recorded in the two-beam condition for **g** = ${111}$. (**a**) The initial stage of stacking faults emission event. (**b**–**e**) A continuous emission of stacking faults from the Σ3{111} boundary. The periodic contrast change was observed during the deformation. (**f**) The adjacent grain shows piled-up dislocations impinged on the Σ3{111} boundary and a local stress concentration field near grain boundary region. (**g**) A magnified view shows another stacking fault nucleated from a different Σ3{111} boundary taken in the two-beam condition for **g** = ${020}$, near a ${[{10}\bar{2}]}_{\mathrm{fcc}}$ zone axis. The fringe contrast changes were indicated by the arrows filled with dots (F_1_, F_2_, F_3_).

Figure 2a–e are selected frames extracted from an in-situ TEM tensile test video (see the original video from the Supplementary Video S1 online) and (f) is an additional BF TEM image taken at the end of the test with holding the applied stress. In the initial stage of deformation, a sequential stacking fault nucleation from a Σ3{111} boundary was observed (a–e). This successive overlapping stacking fault emission event was evident based on the periodic changes in the image contrast and forward moving leading Shockley partial dislocation contrast (e.g., Fig. 2e). Consequently, this sequential stacking fault nucleation will generate the three-layered stacking faults, which is the precursor of a deformation twin. This process is schematically illustrated in Supplementary Fig. S2 in Supplementary information. A localized stress concentration field caused by piled-up dislocations exists on the opposite side of the grain boundary as shown in Fig. 2f. Several secondary slip systems were activated as a result of piled-up dislocations interacted with the Σ3{111} boundary, reflecting the cumulative effect of the residual grain boundary dislocation buildup.

Figure 2g shows a magnified view of a similar stacking fault nucleated from a different Σ3{111} boundary taken in a two-beam condition near a [${10}\bar{2}$]_fcc_ zone axis with the operative reciprocal lattice vector **g** = 020. The two-beam imaging condition exhibits a periodic dark/bright fringe known as alpha-fringe contrast^39^ in this wide stacking fault with few exceptions in the outer most fringes, i.e., F_1,_ F_2,_ and F_3_. A periodic contrast reversal can be observed when the emission of closely spaced overlapping stacking faults lying on parallel (111) planes occurred from a grain boundary because of the phase angle change^40^. Every third set of fringes results in no-contrast because the phase angle α is changed by ± 2/3π every time a single-layered stacking fault passaging by, i.e., α  = 3 ×  ± 2/3π =  ± 2π. The fault scheme—F_1_ represents a single-layered stacking fault while an opposite (bright) contrast of F_2_ corresponds to two-layered stacking fault. The F_3_ showing zero contrast represents the three-layered stacking faults. This image, therefore, supports the nucleation process illustrated in Supplementary Fig. S2 in Supplementary information and we conclude the reason for the periodic contrast reversal in Supplementary Video S1 online is attributed to the layer-by-layer emission of individual leading Shockley partial dislocations lying on adjacent slip planes from the Σ3 boundary.

The formation of three-layered stacking faults on consecutive (111) planes changes the stacking sequence of original FCC of ABCABCABC to ABABCABCA, ABACABCAB and ABACBCABC sequentially. In the first step, an intrinsic stacking fault is formed, i.e., changed the local FCC structure into the HCP one. The subsequent formation of second and third stacking faults are emitted onto one/two atomic layers above the former (111) plane in the second and third steps, respectively. As a result, a deformation twin having two (111) atomic layers was formed, indicated by red dash line in Supplementary Fig. S2 (step-3)). The corresponding stacking fault images combined with each transformation schematic clearly elaborate how the emitted layer-by-layer leading Shockley partial dislocations evolve to a deformation twin.

### High-angle boundaries

**Figure 3:**
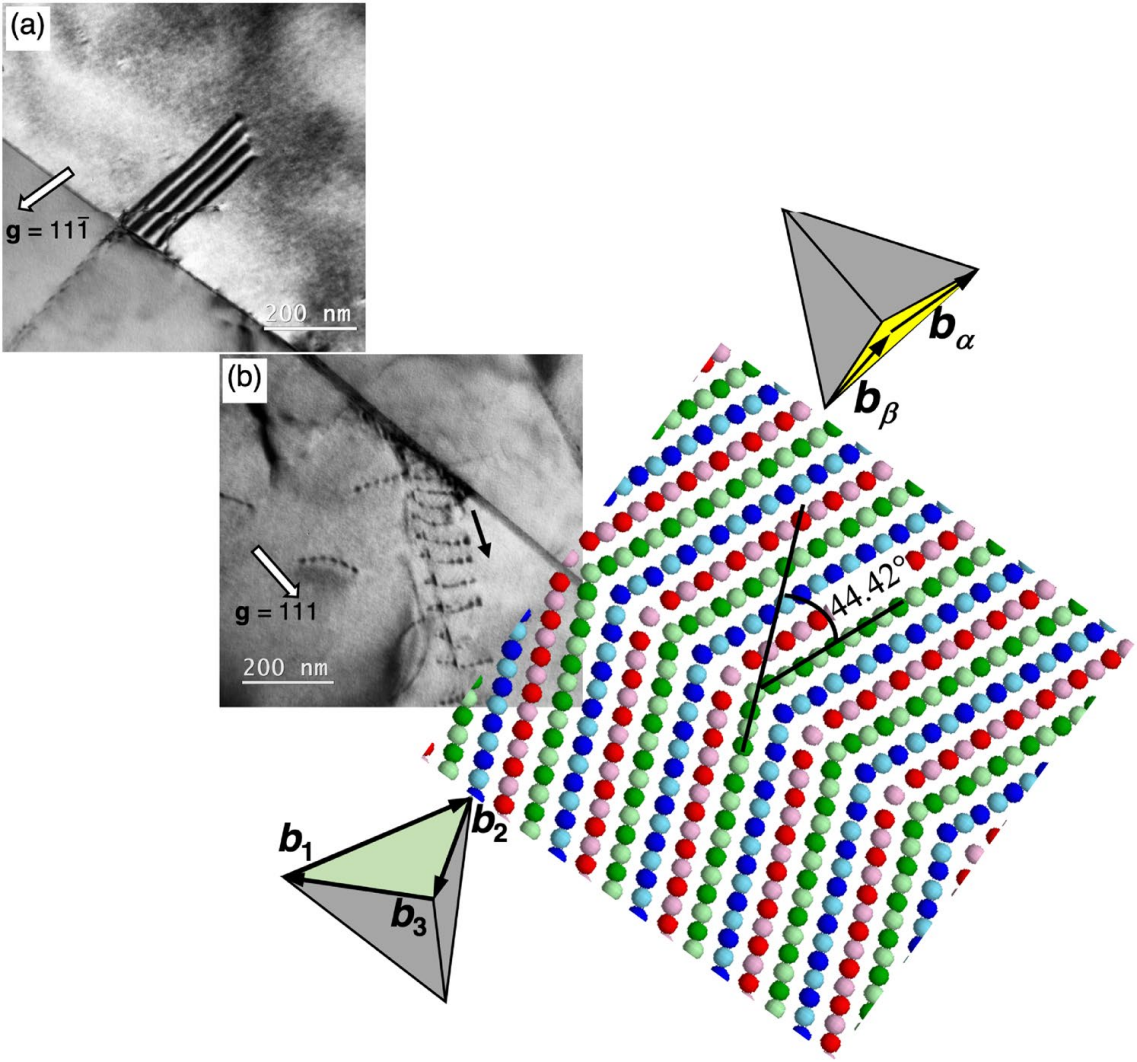
An experimentally observed Σ21 boundary is superimposed onto the [112]Σ21 boundary structure model extracted from the ref.^41^. This Σ21 boundary was defined to have a [112]/44.42° axis/angle pair, which grain-boundary structure is composed of one structural unit A from Σ1 boundary and two structural unit B from Σ11 boundary. The perfect dislocation having Burgers vector of **b**_**1**_ or **b**_**2**_ or **b**_**3**_ was emitted to bottom grain, while the partial dislocation having Burgers vector of **b**_**α**_ or **b**_**β**_ was emitted to the top grain.

A non-Σ3 grain boundary acting as a dislocation source was observed in Fig. 3. These two beam BF-TEM images were taken from the grains on the both side of a high-angle grain boundary. The operative reciprocal lattice vectors were (a) **g** = ${11}\bar{1}$ and (b) **g** = 111 and the engineering strain was 0.02. Both images indicated that dislocations or a stacking fault were nucleated from the grain boundary without the presence of a local stress concentration field near the nucleation site. The grain boundaries in Fig. 3a,b were identified to have a [459]/44.2° axis/angle pair that is basically identical to Σ21 boundary ([112]/44.2°) as shown in the inset atomic structure model^41^. The nucleation of stacking faults in/near the Σ21 boundary could occur without having a local stress concentration field such as dislocation pile-ups in contrast to the case of Σ3{111} boundaries.

**Figure 4:**
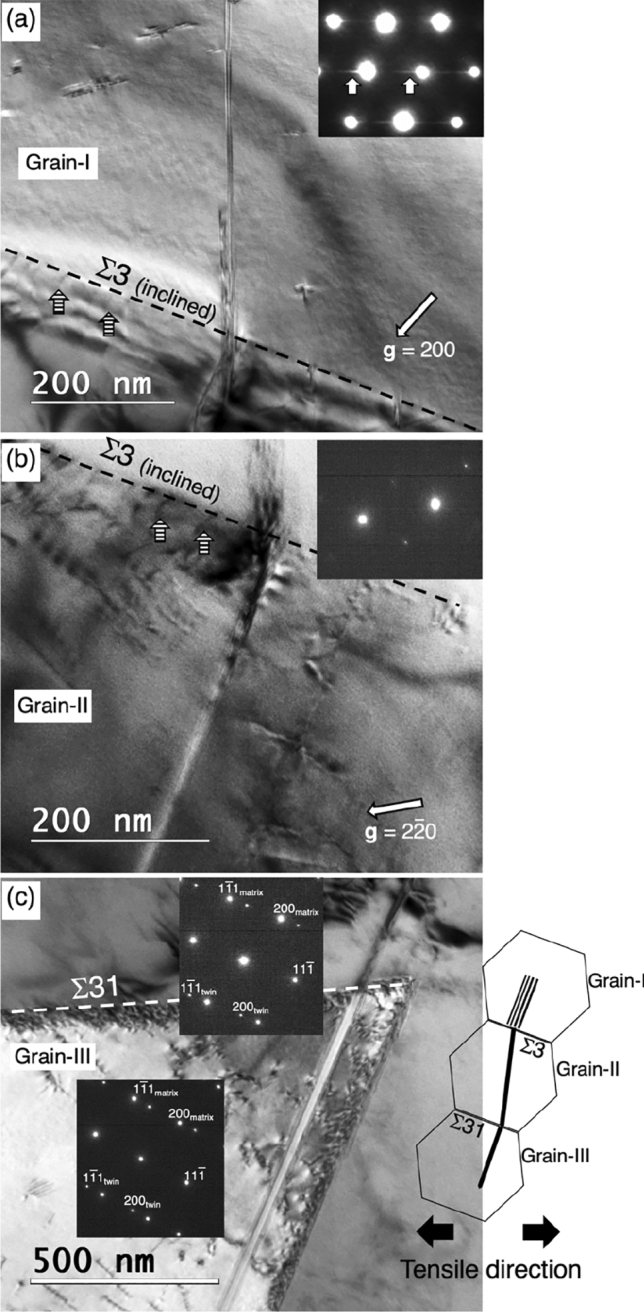
(**a**) A BF TEM image shows a stacking fault in the impingement region of an inclined Σ3{111} boundary. The inset SAED pattern shows faint streaks ascribed to the shape factor of the stacking fault. (**b**) A deformation twin emitted from the Σ31 boundary impinging on the Σ3{111} boundary. Several impingement-induced grain boundary dislocations are indicated by striped arrows. (**c**) Deformation twins were nucleated from a Σ31 boundary. The inset [${0}\bar{1}\bar{1}$] zone axis SAED patterns were taken from each of fine deformation twins. A schematic illustration is provided to indicate the geometrical relationship of two twinned grains (Grain-II and Grain-III) and one grain having a stacking fault (Grain-I).

Figure 4 shows a propagation of deformation twining across two dissimilar grain boundaries. The images were taken at the engineering strain of 0.046. Three grains were separated by two grain boundaries, i.e., a Σ3{111} boundary lies between the grain I and II, whereas a high angle tilt Σ31 boundary separates grains II and III. The twin planes of these deformation twins were identified to be (${11}\bar{1}$) from the corresponding SAED pattern in Fig. 4c. Two deformation twins were attached to the well-defined Σ31 boundary having a [${11}\bar{1}$]/18° axis angle pair. We assume that the Σ31 boundary here serves as a heterogeneous nucleation site for the deformation twins because the coherent Σ3{111} boundary having a stable structural configuration is not energetically favorable to act as a dislocation source^41, 42^ thus it requires a local stress concentration field to nucleate a stacking fault which does not exist here.

On the other hand, the emission of stacking faults in the grain-I could be a result of the deformation twin impingement on the Σ3{111} boundary, which would generate a local stress concentration. This is similar to the stacking faults nucleation associated with dislocation pile-ups against a Σ3{111} boundary (Fig. 1b,d). In response to the localized stress at the grain boundary, the incoming Shockley partial dislocations originated from the Σ31 boundary could dissociate into the grain boundary dislocations which can be seen in Fig. 4b, and Shockley partial dislocations trailing a stacking fault in the adjacent grain-I. The identification of the stacking fault was based on its fringe contrast and weak streaks in SAED pattern in Fig. 4a. It appears that stress localization caused by planar defects impingement at the Σ3{111} boundary could be accommodated by emitting new planar defects to the adjacent grain.

**Figure 5:**
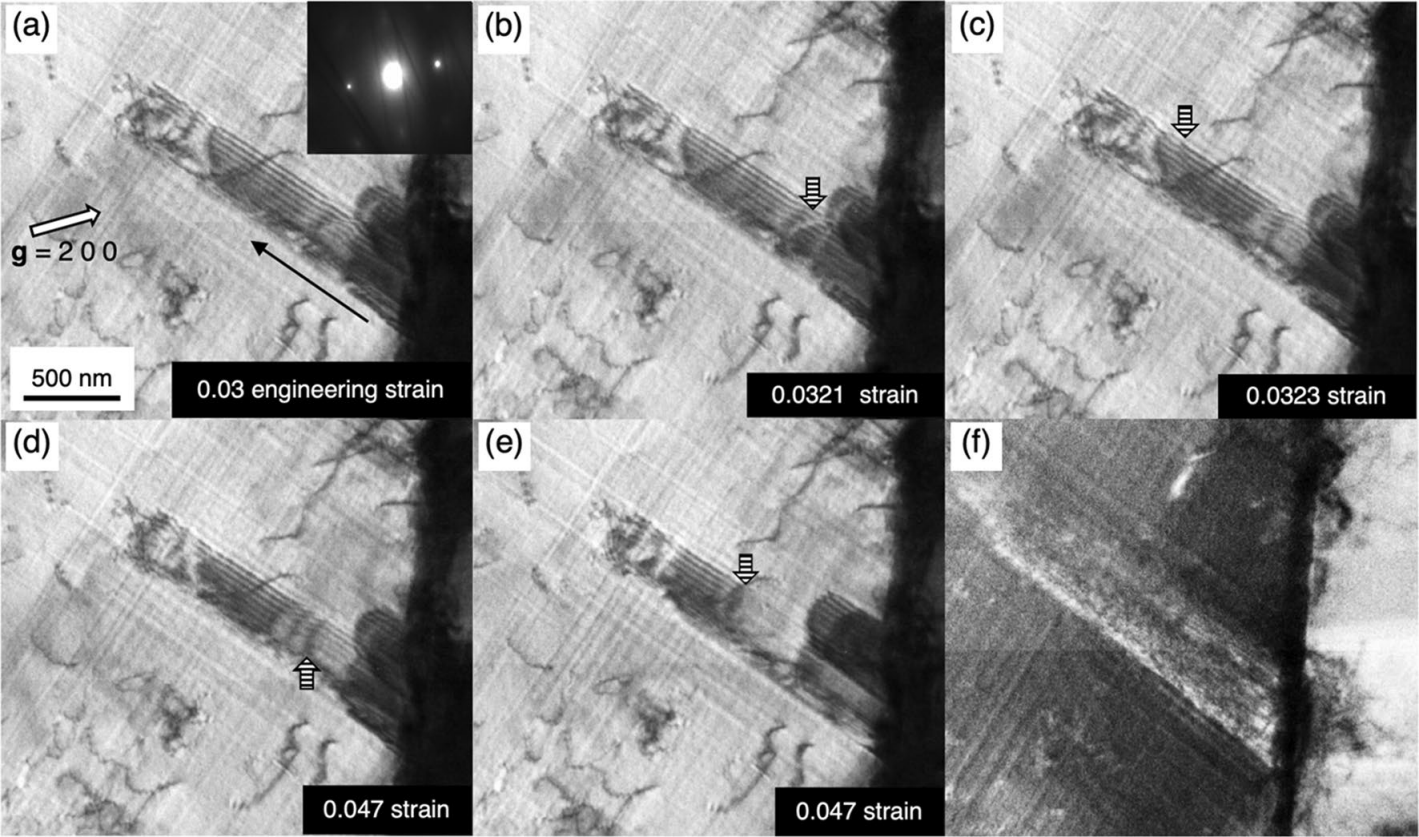
Selected frames of an in-situ deformation TEM test video (see Supplementary Video S2 online) showing near a Σ73 boundary region where the continuous stacking fault emission occurred. The images were recorded in a two-beam condition for **g** = ${200}$. (**a**) An intrinsic stacking fault having the dark outer fringe. (**b**,**c**) A leading partial dislocation emitted from the grain boundary gliding away on an adjacent slip plane, indicated by striped arrow. It results in two stacking faults overlapped (reversal fringe contrast, i.e., white outer fringe). (**d**,**e**) The third leading partial dislocation emitted from the grain boundary into the adjacent fault plane (indicated by the striped arrows) caused a three-layer deformation twin being out of contrast. (**f**) A near grain boundary region of the adjacent grain shows no dislocation pile-ups. A faint contrast induced by the grain boundary itself was visible.

Similar to the Σ21 boundary’s case, a Σ73 boundary where the adjoining lattices are tilted by 41.4° rotation angle about the [010] axis, was found to act as a dislocation source during plastic deformation as shown in the selected frames from an in-situ TEM deformation test video in Fig. 5 (see the original video in Supplementary Video S2 online). The misorientation of this grain boundary was identified from the EBSD analysis implemented prior to the in-situ deformation experiment. The experiment was conducted in a two-beam condition with the operative reciprocal lattice vector **g** = 200 near a [001]_fcc_ zone axis, and at a slightly higher magnification than Fig. 2 to clarify the details of the nucleation behavior (see Supplementary Video S2 online). In Fig. 5a, an intrinsic stacking fault with a dark outer fringe was observed. During the plastic deformation, another leading Shockley partial dislocation (striped arrow) on the adjacent parallel fault plane was generated from the grain boundary (Fig. 5b). As the leading Shockley partial dislocation continuously glide away from grain boundary (Fig. 5c), the outer fringe contrast turned from dark to bright, i.e., two stacking faults were overlapped. Soon after that, the next emission of leading Shockley partial dislocation made the fringe contrast none (Fig. 5d,e) due to an effective transition vector value R = 3 × 1/3 (111) is equivalent to that of a perfect lattice. These successive emission events are fast and a three-layered stacking fault, the precursor of deformation twin, formed as a consequence. Our two in-situ TEM experiments indicate that the mechanism of the deformation twin precursor is identical in both Σ3{111} and Σ73 boundaries; both are by the sequential stacking fault emission mechanism.

## Discussion

The deformation behavior of this medium-SFE TWIP steel at/near grain boundaries appears to be similar to that in low SFE (SFE ~ 12 mJ/m^2^) TWIP steels rather than the high SFE (SFE ~ 85 mJ/m^2^) counterparts^6^, i.e., the main carrier of plastic deformation is deformation twins and stacking faults rather than dislocation in these areas, and the deformation twin nucleation was associated with both perfect and Shockley partial dislocations. Our observations indicate that the deformation twins having two (111) atomic layers were formed by a sequential emission of stacking faults from grain boundaries, which is different from the conventional Mahajan-Chin three-layer mechanism^19^.

Piled-up dislocations at a grain boundary could trigger twin formation to accommodate the localized stress concentration^43–46^, and annealing twin (Σ3{111}) boundary appears to be the most favorable deformation twin nucleation site especially those in a grain which a ⟨111⟩_fcc_ orientation is parallel to the tensile axis^20^. The piled-up dislocations assisted deformation twin nucleation at a Σ3{111} boundary can be a two-step process, i.e., Step-1: a stress concentration relaxation event at the annealing twin boundary when a group of dislocations are plied-up, which followed by Step-2: emitting stacking faults on successive slip planes that will be evolving into a deformation twin.

**Figure 6:**
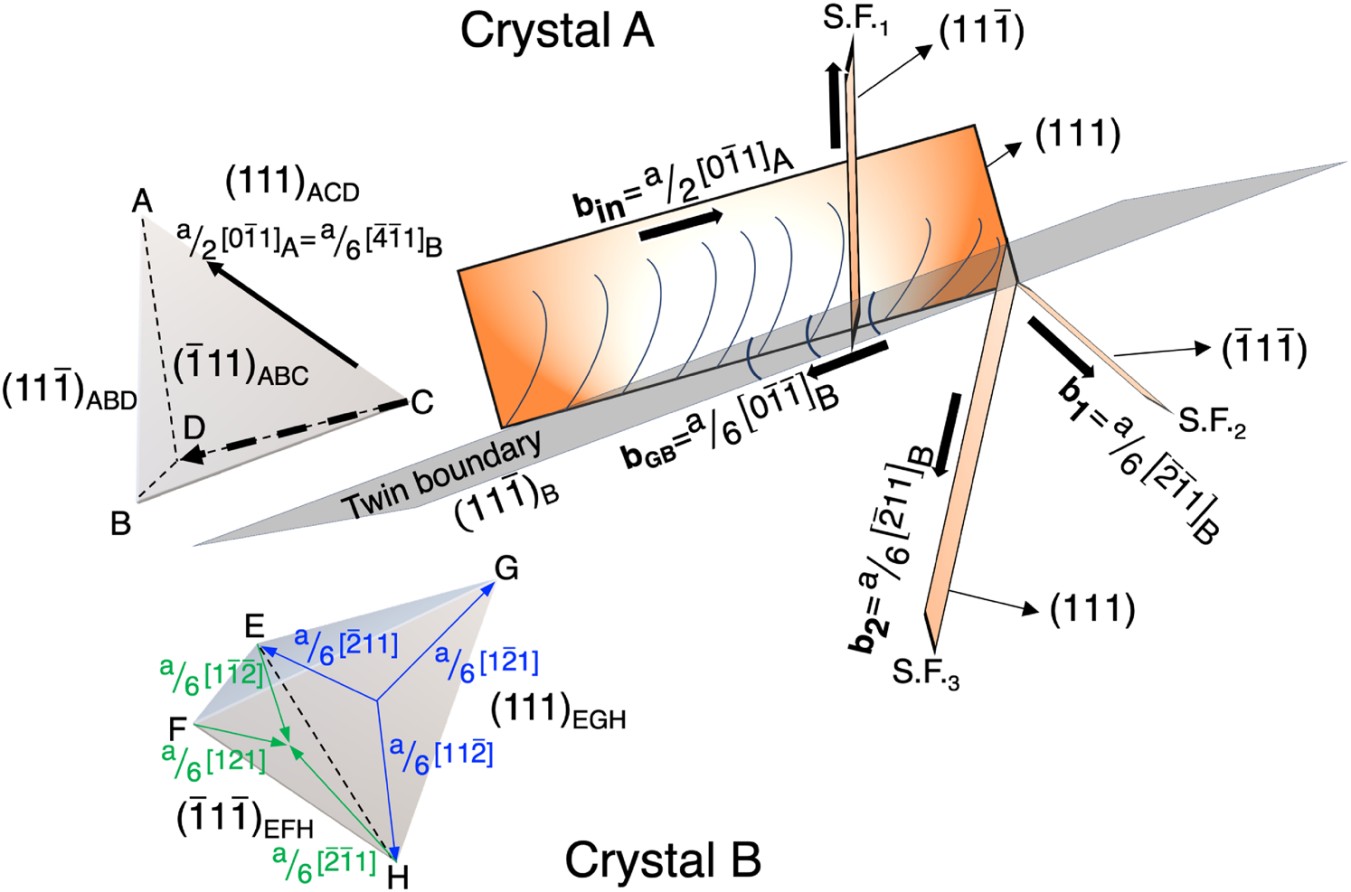
A schematic illustration shows the precursors of deformation twin are initiated at a near grain boundary region as a result of the impingement between piled-up dislocations and an annealing twin boundary. The stacking fault S.F._1_ is formed by a cross-slip dislocation reaction. S.F._2_ and S.F._3_ are formed by a grain boundary stress relaxation reaction. The Burgers vector of incoming dislocations ${\text{b}}_{\text{in}}$ was designated to be ${\text{a}}/{2}{\text{[0}\bar{1}\text{1]}}_{\text{A}}$ or ${\text{a}}/{6}{\text{[}\bar{4}\bar{1}\text{1]}}_{\text{B}}$ after a proper matrix transformation, whereas the Burgers vectors of b_1_ ${\text{and}}$ b_2_ were assumed to be two Shockley partial dislocations (${\text{a}}/{6}{\left[\bar{2}\bar{1}{\text{1}}\right]}_{\text{B}}$ and ${{\text{a}}/{6}\left[\bar{2}{\text{11}}\right]}_{\text{B}})$ that are emitted to minimize the magnitude of Burgers vector of grain boundary dislocations.

The first step can be explained by the dislocation—Σ3{111} boundary interaction schematically summarized in Fig. 6, which is basically representing the case in Fig. 1c. Incoming dislocations interacting with a Σ3{111} boundary leave more than one stacking fault on the both sides of the Σ3{111} boundary. It has been a generally accepted understanding that a coherent Σ3{111} boundary is not acting as a proactive dislocation source because of its coherent atomic arrangement^41, 42, 47–51^, which is different from grain boundaries consisting of long-period structure units. However, the interaction between Σ3{111} boundary and incoming dislocations (**b**_**in**_) could trigger different reactions at the grain boundary. As seen in Fig. 6, dislocations approaching the Σ3{111} boundary could make cross-slip and transfer to the adjacent grain only when the incoming dislocations contain a specific Burgers vector, illustrated as $\overline {{\text{CD}}}$ in the Thompson tetrahedron ABCD. Otherwise, the incoming dislocations (**b**_**in**_) would be obstructed by the Σ3{111} boundary and inevitably encounter an energy barrier of which strength is related to the SFE of the alloy, thus, a certain amount of energy is required to compress Shockley partial dislocations at the boundary. The process of compressing a pair of Shockley partial dislocations into a perfect dislocation on the Σ3{111} boundary gradually builds up a large stress field in/near the boundary regions, which obstructs the entry of additional incoming dislocations thereby stimulating the dislocation pile-ups and will lead to two possible deformation twinning behavior. First, the incoming dislocations may start to dissociate into Shockley partial dislocations on the conjugate slip planes of the original grain due to the alloy’s relatively low SFE characteristic, which could be the case appeared in Figs. 1c,d and 2f. The nucleation of stacking faults of S.F.1 in the original grain is the result of dislocation dissociation, i.e., the incoming dislocations dissociate into the Shockley partial dislocations lying on the conjugate slip plane by the Cohen-Weertman or Fujita-Mori cross-slip twinning mechanism, suggesting that the deformation twinning behavior could occur in the vicinity of grain boundaries.

Second, the leading piled-up dislocation attached to the Σ3{111} boundary is required to dissociate its Burgers vector into multiple components in order to decrease the dislocation pile-up caused elastic strain energy in the neighbor grain region. A theoretical study of dislocation—Σ3{111} boundary interaction in aluminum, copper, and nickel by molecular dynamics simulation indicates that the outgoing slip mechanism to an adjacent grain, associated with dislocation decomposition in a Σ3{111} boundary, depends on the relationship between the mechanical factor, i.e., resolved driving force, and the material factor, i.e., lattice resistance for re-nucleating partial dislocations determined by the plane fault energies^35^. Although the actual internal and external stress components around the Σ3{111} boundary are not measurable easily, the outgoing Shockley partial dislocations (**b**_**1**_ and **b**_**2**_) into Crystal-B along with S.F.2 and S.F.3 (Fig. 6) must receive a larger resolved driving force than that on the Shockley partial dislocation on the Σ3{111} boundary due to the stress concentration by the dislocation pile-up. It suggests that creating partial dislocations into an adjacent grain seems to be a more favorable path in response to the localized stress field induced by the dislocation pile-ups at the Σ3{111} boundary.

Minimizing the magnitude of Burgers vector of the residual dislocations left in a grain boundary is generally believed to be the most dominant criterion to determine which slip system will be activated from the grain boundary in response to a local stress concatenation^33, 52–54^. After a strong dislocation—grain boundary interaction, the grain boundary dislocations having a large magnitude of Burgers vector would be unfavorable, thus the dissociated leading Shockley partial dislocations were emitted from the grain boundary in response to the unstable grain boundary structure. The Burgers vector of residual grain boundary dislocations can be determined by the following relationship:

1 $${\mathbf{b}}_{\mathbf{G}\mathbf{B}}={\mathbf{b}}_{\mathbf{in}}-{\mathbf{b}}_{\mathbf{out}},$$

where **b**_**GB**_ is the difference between the incoming and outgoing dislocations’ Burgers vectors, **b**_**in**_ and **b**_**out**_, respectively. The **b**_**in**_, a/2 [${0}\bar{1}{\text{1}}$]_A_ in Crystal-A or a/6 [$\bar{4}\bar{1}{\text{1}}$]_B_ after a proper coordinate transformation in Crystal-B, can be characterized by the **g⋅b** criterion^55^. On the other hand, the **b**_**out**_ in the adjacent grain cannot be resolved but the **b**_**1**_ and **b**_**2**_ must be the Shockley partial dislocations lying on ($\bar{1}{\text{1}}\bar{1}$) and (111), respectively. i.e., ± a/6 [121], ± a/6 [$\bar{2}\bar{1}{\text{1}}$], or ± a/6 [${1}\bar{1}\bar{2}$] for **b**_**1**_, and ± a/6 [${1}\bar{2}{\text{1}}$], ± a/6 [$\bar{2}{\text{11}}$], or ± a/6 [${11}\bar{2}$] for **b**_**2**_. While 36 possible combinations for the emitted Shockley partial dislocations are expected, two possible reactions can be considered by assuming the emitted Shockley partial dislocations would minimize the magnitude of Burgers vector of grain boundary dislocation:

2 $${\mathbf{b}}_{\mathbf{G}\mathbf{B}}={\raise0.7ex\hbox{${\rm{a}}$} \!\mathord{\left/ {\vphantom {{\rm{a}} 6}}\right.\kern-\nulldelimiterspace} \!\lower0.7ex\hbox{$6$}}{\text{[}\bar{4}\bar{1}\text{1]}}_{\text{B}}-\left\{{\raise0.7ex\hbox{${\rm{a}}$} \!\mathord{\left/ {\vphantom {{\rm{a}} 6}}\right.\kern-\nulldelimiterspace} \!\lower0.7ex\hbox{$6$}}{\left[\bar{1}\bar{2}\bar{1}\right]}_{\text{B}}\text{+}{\raise0.7ex\hbox{${\rm{a}}$} \!\mathord{\left/ {\vphantom {{\rm{a}} 6}}\right.\kern-\nulldelimiterspace} \!\lower0.7ex\hbox{$6$}}{\left[\bar{2}{\text{11}}\right]}_{\text{B}}\right\}={\raise0.7ex\hbox{${\rm{a}}$} \!\mathord{\left/ {\vphantom {{\rm{a}} 6}}\right.\kern-\nulldelimiterspace} \!\lower0.7ex\hbox{$6$}}{\left[\bar{1}{\text{01}}\right]}_{\text{B}}$$

3 $${\mathbf{b}}_{\mathbf{G}\mathbf{B}}={\raise0.7ex\hbox{${\rm{a}}$} \!\mathord{\left/ {\vphantom {{\rm{a}} 6}}\right.\kern-\nulldelimiterspace} \!\lower0.7ex\hbox{$6$}}{\text{[}\bar{4}\bar{1}\text{1]}}_{\text{B}}-\left\{{\raise0.7ex\hbox{${\rm{a}}$} \!\mathord{\left/ {\vphantom {{\rm{a}} 6}}\right.\kern-\nulldelimiterspace} \!\lower0.7ex\hbox{$6$}}{\left[\bar{2}\bar{1}{\text{1}}\right]}_{\text{B}}\text{+}{\raise0.7ex\hbox{${\rm{a}}$} \!\mathord{\left/ {\vphantom {{\rm{a}} 6}}\right.\kern-\nulldelimiterspace} \!\lower0.7ex\hbox{$6$}}{\left[\bar{2}{\text{11}}\right]}_{\text{B}}\right\}={\raise0.7ex\hbox{${\rm{a}}$} \!\mathord{\left/ {\vphantom {{\rm{a}} 6}}\right.\kern-\nulldelimiterspace} \!\lower0.7ex\hbox{$6$}}{\left[{0}\bar{1}\bar{1}\right]}_{\text{B}}$$

The **b**_**GB**_ = a/6 [$\bar{1}{\text{01}}$]_B_ in Eq. (2) contains the component perpendicular to the twin boundary (${11}\bar{1}$), whereas the **b**_**GB**_ = a/6 [${0}\bar{1}\bar{1}$]_B_ in Eq. (3) could freely slip on the twin plane. Thus, the Eq. (3) would be the most possible reaction in response to accommodate the incoming dislocations at the grain boundary.

Now, the second step of the deformation twin nucleation, consisting of the sequential emission of stacking faults on the successive slip planes, can be explained by the ratio of the intrinsic stacking fault energy and unstable stacking fault energy, γ_isf_/γ_usf_^56^. The movement of leading Shockley partial dislocation requires to overcome the energy barrier γ_usf_, while trailing Shockley partial dislocation encounters a much lower energy barrier associated with γ_usf_–γ_isf_. The effect of trailing Shockley partial dislocations on deformation twinning can be mitigated when the difference between γ_isf_ and γ_usf_ is large enough to promote the formation of wide stacking faults as the deformation proceeded. The γ_isf_ of our alloy is around 40 mJ/m^2^ while the γ_usf_ is hard to estimate experimentally. However, the formation of stacking fault in the neighboring slip planes would experience the energy barrier which value may be similar to γ_usf_^5, 57, 58^. A universal energy relationship of the planar fault energy barriers in many FCC metals would be:

4 $${\upgamma}_{\text{utf}} \cong {\raise0.7ex\hbox{$1$} \!\mathord{\left/ {\vphantom {1 2}}\right.\kern-\nulldelimiterspace} \!\lower0.7ex\hbox{$2$}} \cdot {\upgamma}_{\text{isf}} + {\upgamma}_{\text{usf}},$$

where γ_utf_ is the energy needed to transform an intrinsic stacking fault to an extrinsic stacking fault. For low SFE metals, the 1/2 γ_isf_ term becomes negligible so that the γ_utf_ will be similar to γ_usf_. The minimum energy path for continuous generation of planar faults such as intrinsic/extrinsic stacking faults and twins on neighboring slip planes could be achievable as long as the energy barriers γ_usf_ and γ_utf_ are conquerable. Our results indicate that dislocations pile-up is required to conquer the energy barriers at Σ3{111} boundaries whereas different types of boundaries could lower the energy barriers by its structural characteristics.

The deformation twinning behavior in high-sigma-value boundaries (Σ21, Σ31 and Σ73) indicate that these boundaries could act as a dislocation source thus stacking faults and deformation twins can be nucleated directly from these in response to tensile deformation, which is different from what was observed in Σ3{111} boundary. Thus, the grain boundary character undoubtedly affects the deformation twin nucleation behavior, in addition to the SFE and geometrical grain orientation relative to the loading axis.

A grain boundary with a specific orientation can be constructed by combing delimiting grain boundaries^59^; for example, a [001]Σ17 tilt boundary can be composed from one structural unit from Σ1 boundary and two units from Σ5 boundary, which is designated to be the |ABB| periodic structure^60^. The grain boundaries constructed by long-periodic structural units usually have a higher grain boundary energy^41, 42, 51, 61^. Computational studies^41, 60, 61^ demonstrate that the [112]Σ21, [${11}\bar{1}$]Σ31, and [001]Σ73 tilt boundaries can be described to be long-period boundaries, i.e., the extrinsic dislocations can be introduced to the boundaries to accommodate the misorientation angle deviated from the delimiting boundaries. The delimiting grain boundaries show the weakest trend of acting as a dislocation source^41^ and the trend becomes even stronger as the boundary structure deviating from the delimiting one. Thus, the [112]Σ21 boundaries bordering the stable [112]Σ11 delimiting boundary could eventually transform to a relatively stable boundary structure by emitting dislocations and minimizing the magnitude of Burgers vector of grain boundary dislocations. As schematically shown in Fig. 3, a [112]Σ21 boundary emits dislocations to both top and bottom grains. In this process, a perfect dislocation having Burgers vector of **b**_**1**_ or **b**_**2**_ or **b**_**3**_ will be emitted to the bottom grain, while a Shockley partial dislocation having Burgers vector of **b**_**α**_ or **b**_**β**_ will be emitted to the upper grain. The rotation axis of [112]Σ21 boundary is aligned well with the dislocation lines of Shockley partials and perfect dislocations. Since the grain boundary structure transition utilizes the Burgers vector of grain boundary dislocations associated with the misorientation of that particular grain boundary, this rotation axis—dislocation line alignment observed in the top grain makes dislocation emission easier; this event is the same as that observed in a bicrystal model with a Σ21 boundary under a uniaxial loading test by molecular dynamics simulation^41^. On the other hand, the emitted dislocation line in the bottom grain is not parallel to the rotation axis, so that the dislocations are emitted not by using grain boundary dislocations, but by using free volume that does not exist in coherent boundaries such as Σ3{111}. This asymmetrical phenomenon is probably caused by the complicated multiaxial stress state around the grain boundary.

An atomic structure model was built to reproduce the geometrical relationship between Grains-I and -II in Fig. 4a and to evaluate the Schmid factor for possible slip systems in this particular geometry (see Supplementary Fig. S3). The Schmid factor for the stacking fault in the Grain-II is calculated to be 0.244, which is the third largest among the enabled partial dislocations in the Grain-II and would be reasonable when the [${11}\bar{1}$]Σ31 acts as the dominate dislocation source. The Schmid factor for the stacking fault nucleated from the Σ3{111} boundary to the Grain-I is relatively low. Since the slip system having the largest Schmid factor was not activated, the propagation of the slip from the Grain-II could play a critical role in this particular case to propagate plastic deformation. Also notice that the possible direction of the Burgers vector nucleated from the Σ3{111} boundary to the Grain-I is almost opposite direction of the Burgers vector approaching to the Σ3{111} boundary in the Grain-II; hence, the residual Burgers vector at the Σ3{111} boundary after the dislocation transfer becomes large under the applied tensile stress. These considerations suggest that the applied tensile stress itself is not enough to make the slip propagation from the Grain-II to Grain-I, thus the stacking fault formation at the Σ3{111} boundary requires additional driving force such as local stress concentration.

Shockley partial dislocation emission was also observed in the $[{11}\bar{1}]$Σ31 boundary in Fig. 4b and the [001]Σ73 boundary in Fig. 5. The complicated multiaxial stress applied on these long-period grain boundary structures appear to stimulate the $[{11}\bar{1}]$Σ31 and [001]Σ73 boundaries act as a dislocation source, but whether the grain boundary structure transition involved in the dislocation emission process or free-volume assisted dislocation emission process cannot immediately be confirmed. Since the rotation axis of ⟨111⟩ and ⟨001⟩ boundaries have no direct relation with the dislocation line direction, the mechanism of dislocation emission for ⟨111⟩ and ⟨001⟩ boundaries would be different from that for Σ21 boundary. However, there is still a possibility that these boundaries also utilize their grain boundary dislocations for dislocation emission, or conquering energy barriers for deformation twin nucleation.

## Conclusions

In this study, how grain boundary misorientation influences deformation twinning nucleation mechanism at grain boundaries in an Fe-31Mn-3Al-3Si TWIP steel was investigated by in-situ deformation TEM experiments followed by detailed crystallographic analysis.

1. Deformation twin nucleation at a Σ3{111} boundary occurs when a local stress concentration field at or near the boundary exceeds a twinning stress. Σ3{111} boundary can act as a strong barrier against dislocations and planar defects motion thus a local stress concentration field needs to be introduced by dislocations or planar defects piled-up against the Σ3{111} boundary. The influence of the barrier effect causing the deformation twin nucleation depends on the characteristics of the incoming dislocations to a Σ3{111} boundary. The deformation twin nucleation at a Σ3{111} boundary would not occur when the incoming dislocations made slip transfer across it.
2. Deformation twin nucleation at high angle grain boundaries such as Σ21 or Σ31 was not accompanied with a local stress concentration field caused by structural defects. Our microstructure observations indicate that high angle grain boundaries would spontaneously emit stacking faults because their long-period structure units contain lattice dislocation components accommodating the misorientation angle deviated from the delimiting boundaries.
3. Successive layer-by-layer stacking fault emission is found to be the deformation twin nucleation mechanism in this study at both low and high sigma value grain boundaries. Deformation twins having two (111) atomic layers were formed by a sequential emission of leading Shockley partial dislocations from grain boundaries in our in-situ TEM experiments, which is different from the conventional Mahajan-Chin three-layer mechanism.

## Materials and methods

The chemical composition of the alloy is 31.0 Mn, 3.0 Al, 3.0 Si, 0.005 C, 0.004 N, 0.012 S (mass %) and balance Fe. As-received alloy was a hot-forged sheet with 12 mm thickness. Multi-pass cold rolling to 1 mm thick (92% rolling reduction in thickness) was conducted followed by a heat treatment at 950 °C for 15 min. The sample with average grain size of 15.4 μm then was sectioned to a specific dimension, 13 × 2 mm rectangular piece, and thinned mechanically from 1 mm to 150 μm. The foils were tensile-deformed to 0.01, 0.02 and 0.046 engineering strain respectively using a SEM testing stage (Kammrath and Weiss Module 5000N) at a strain rate of 4.6 × 10^–4^ s^−1^ at room temperature.

The samples for TEM analysis were cut from the center of tensile-deformed samples to a specific dimension, 2 × 2 mm square-shape, and then mechanically thinned to 70 μm thick. Thinning to electron transparency was achieved by using a twin-jet electropolisher (E.A. Fischione Model 110) with a 95% acetic acid and 5% perchloric acid electrolyte maintained at 17 °C and the applied voltage, 38 V. The transmission electron microscopy was then performed using a JEOL 2100 TEM operated at 200 kV.

The sample for in-situ deformation tests in TEM were prepared as same as the samples prepared for TEM analysis. The square-shape specimen was fixed on a cartridge-type blade on a SATO Holder Duo (Mel-Build Co.)^62^. The strain rate in this study was controlled at approximately 6.7 × 10^–5^ s^−1^. In-situ tensile experiments were performed on a FEI Titan 300 TEM in the bright field mode, operated at 300 kV. Videos were recorded using Gatan Orius SC200D camera and Digital Micrograph with the high-resolution streaming video plug-in.

## Supplementary Information

Supplementary Information 1.

Supplementary Video S1.

Supplementary Video S2.
