# Supplementary material for: A correlation between grain boundary character and deformation twin nucleation mechanism in coarse-grained high-Mn austenitic steel
Source: Sci Rep. 2021 Apr 19;11:8468. doi: 10.1038/s41598-021-87811-w (PMC8055962; doi:10.1038/s41598-021-87811-w)
Supplement: Supplementary file 1 — Supplementary Information 1. [file 41598_2021_87811_MOESM1_ESM.pdf]

A Correlation Between Grain Boundary Character and Deformation Twin Nucleation Mechanism in Coarse-grained High-Mn Austenitic Steel

Chang-Yu Hung[a,*], Yu Bai[b], Tomotsugu Shimokawa[c], Nobuhiro Tsuji[b,d], Mitsuhiro Murayama[a,e*]

[a] Department of Materials Science and Engineering, Virginia Tech, Blacksburg, VA 24061, USA
[b] Department of Materials Science and Engineering, Kyoto University, Yoshida-honmachi, Sakyo-ku, Kyoto 606-8501, Japan
[c] Department of Mechanical Engineering, Kanazawa University, Kanazawa, Ishikawa, 920-1192, Japan
[d] Elements Strategy Initiative for Structural Materials, Kyoto University, Yoshida-honmachi, Sakyo-ku, Kyoto 606-8501, Japan
[e] Institute for Materials Chemistry and Engineering, Kyushu University, Kasuga, Fukuoka 816-8580, Japan

***Corresponding authors**
Chang-Yu Hung (changyu1@vt.edu) TEL: 540-231-0466, FAX 540-231-1963
Mitsuhiro Murayama (murayama@vt.edu) TEL: 540-231-9470, FAX 540-231-1963

Contact information of authors

Chang-Yu Hung	E-mail: changyu1@vt.edu
Yu Bai,	E-mail: bai.yu.6m@kyoto-u.ac.jp
Tomotsugu Shimokawa	E-mail: simokawa@se.kanazawa-u.ac.jp
Nobuhiro Tsuji	E-mail: nobuhiro-tsuji@mtl.kyoto-u.ac.jp
Mitsuhiro Murayama	E-mail: murayama@vt.edu

Grain misorientation measurement

A grain boundary can be defined by a misorientation angle and an axis (axis/angle pair) of grains on both side of the grain boundary. The Seyring's method[1] was taken here as a reference. In this study, to determine the orientation relationship, the orientation matrix with respect to the reference coordinate system of each grain has to be determined from its electron diffraction pattern. First, a double-tilt TEM holder was used to tilt the sample to a certain degree, which would bring both grains into its particular zone axis. It will be verified that zone axes characterized in the neighboring grains at similar stage tilts could be mapped into each other using a transformation matrix. Then, the diffraction pattern frame ($X_p\, Y_p\, Z_p$) was defined with the Z-axis parallel to the zone axis, the X-axis parallel to any indexed diffraction g-vector, and the Y-axis parallel to the zone−axis × g, which can be expressed as followed:

$$\begin{bmatrix} X_p \\ Y_p \\ Z_p \end{bmatrix} = \begin{bmatrix} g \\ \text{zone axis} \times g \\ \text{zone axis} \end{bmatrix} \quad (1)$$

A reference frame ($X_R\, Y_R\, Z_R$), which lies on fluorescent screen and only differs from $\begin{bmatrix} X_p \\ Y_p \\ Z_p \end{bmatrix}$ by a right-handed rotation of φ about zone axis, where φ is the angle between X_p and X_R. The corresponding rotation matrix is

$$R = \begin{bmatrix} \cos\varphi & \sin\varphi & 0 \\ -\sin\varphi & \cos\varphi & 0 \\ 0 & 0 & 1 \end{bmatrix} \quad (2)$$

By combining the $\begin{bmatrix} X_p \\ Y_p \\ Z_p \end{bmatrix}$ and R, the matrix equivalent to the rotation from crystal to reference can be expressed as:

$$\begin{bmatrix} X_R \\ Y_R \\ Z_R \end{bmatrix} = R \cdot \begin{bmatrix} X_p \\ Y_p \\ Z_p \end{bmatrix} \quad (3)$$

In order to be consistent to the definition made by Bunge [2], the transpose of $R \cdot \begin{bmatrix} X_p \\ Y_p \\ Z_p \end{bmatrix}$ equivalent to grain orientation matrix M that represents the rotation from reference to crystal frame. Finally, the misorientation matrix of two neighboring grains can be determined from their orientation matrix M_1 and M_2:

$$M_{12} = M_2^{-1} * M_1 \quad (4)$$

M_{12} then can be transformed into an axis-angle pair with a proper transformation.

[1] Seyring, M., Song, X. & Rettenmayr, M. Advance in orientation microscopy: Quantitative analysis of nanocrystalline structures. *ACS Nano* **5**, 2580–2586 (2011).

[2] Engler, O; Randle, V. *Introduction to Texture Analysis: Macrotexture, Microtexture and Orientation Mapping, second ed. Taylor & Francia, CRC Press, USA* (2008).

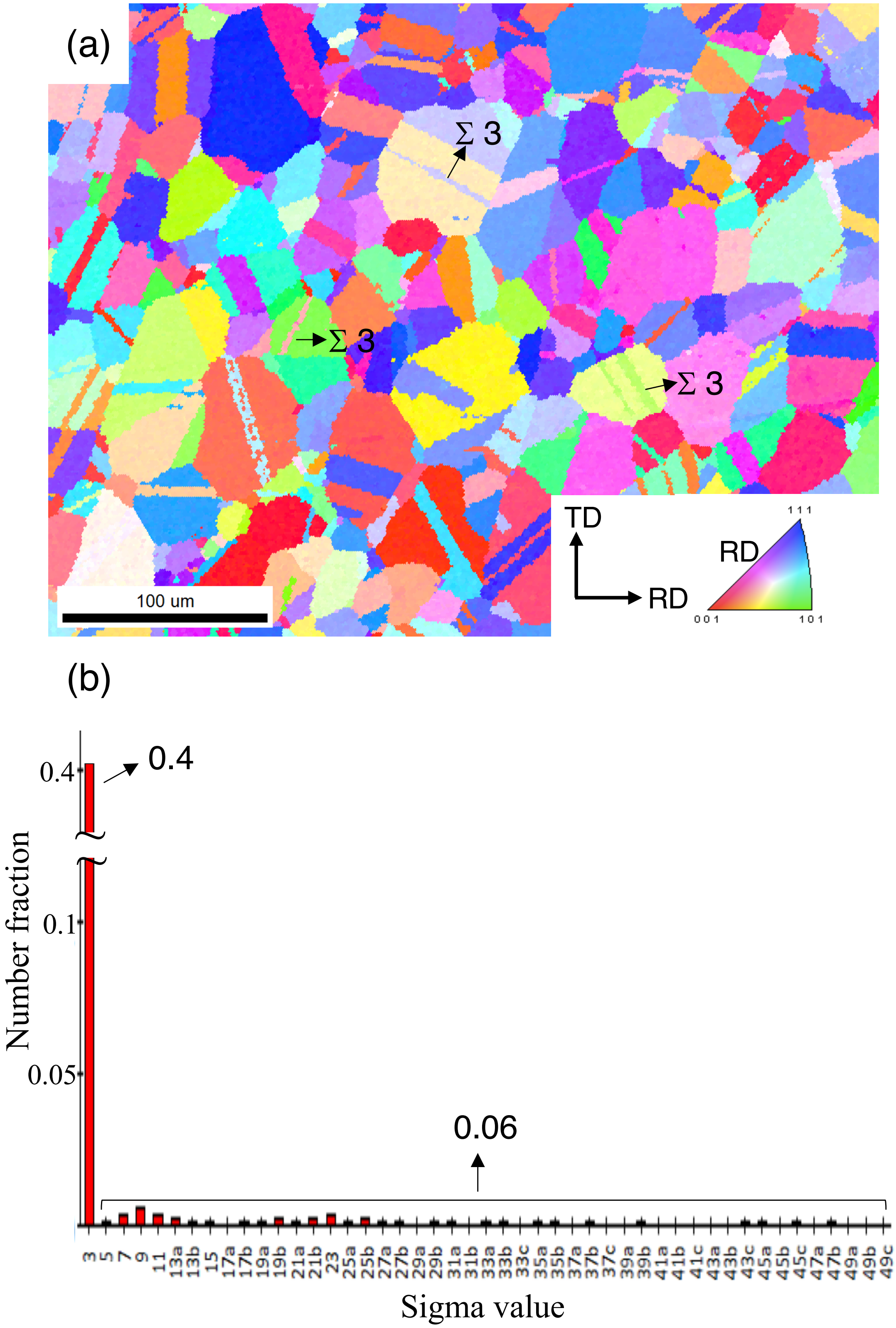

Fig. S1. The grain orientation of an as-received Fe-31Mn-3Al-3Si TWIP steel derived from EBSD. (a) An inverse pole figure (IPF) map indicates a fully recrystallized austenite single-phase structure. The crystallographic orientation is represented as parallel to sample rolling direction; three representative Σ3 boundaries identified to have 111 / 60° axis/angle pairs are indicated by black arrows. (b) The population of coincidence site lattice (CSL) boundaries by taking more than 200 grain boundaries into account. The number fraction is defined to be the ratio of the number of a CSL grain boundary to the total number of grain boundaries; 0.4 for Σ3 boundaries and 0.06 for the rest of CSL boundaries ranging from Σ5 to Σ49 sigma-value boundaries.

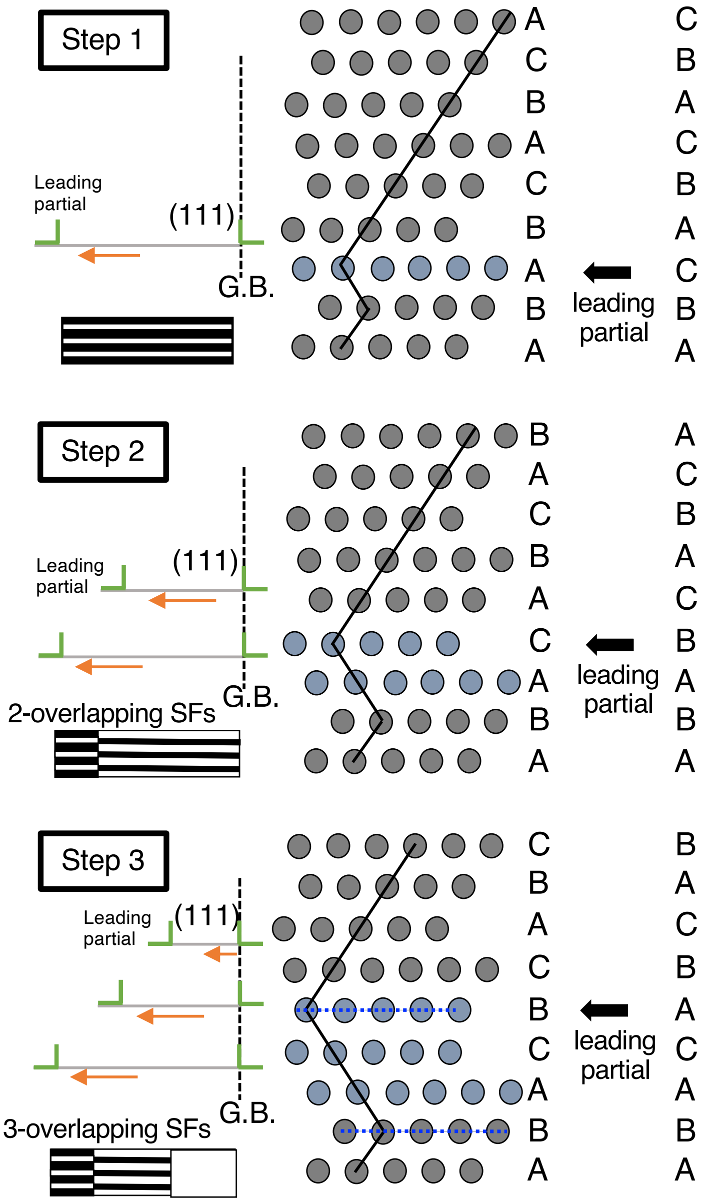

Fig. S2. Schematic illustrations show how fringe contrast reversal occurs by the successive layer-by-layer stacking fault emission. Since the planar defect was formed on a {111} close-packed slip plane, the value of translation vector R could be a 1/3 {111} type, leading the phase angle for the F_1, a = 4/3π equivalent to a = -2/3π, to a dark outer fringe. When a second stacking fault was displaced on an adjacent fault plane, an extrinsic stacking fault would be formed with a phase angle, a = 2/3π, which F_2 showed a reversal contrast when compared with F_1. The presence of no-contrast F_3 is ascribed to three-layered stacking faults with an effective R = 3 × 1/3 (111), which acts like a perfect lattice vector and can therefore appear to give $2\pi g \cdot R = 0$.

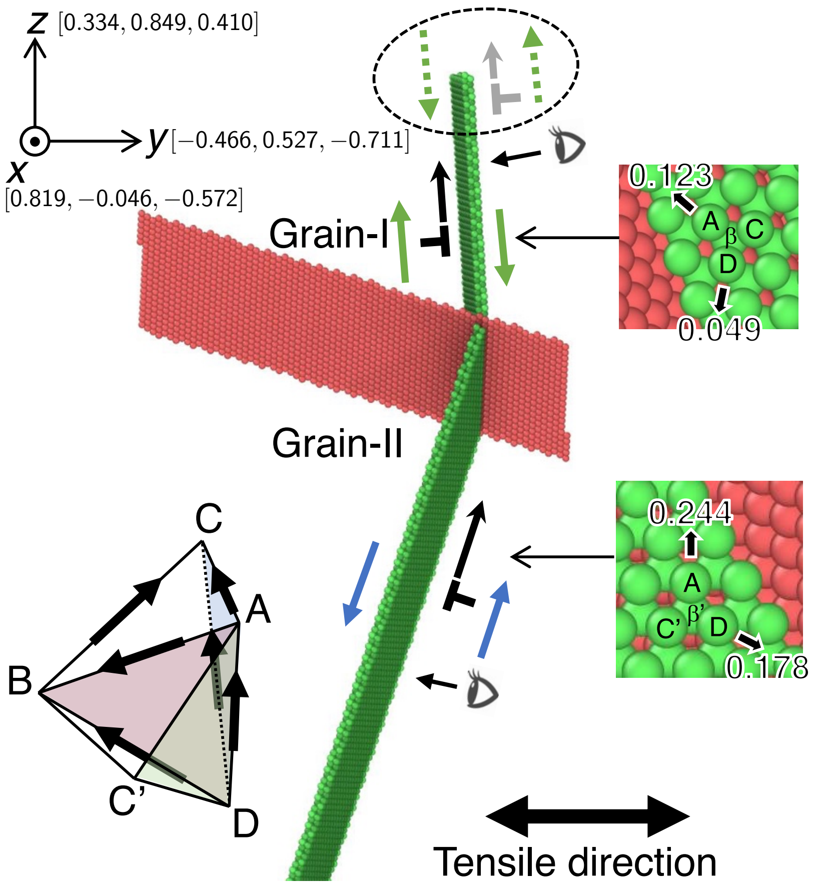

Grain I						
plane	perfect	SF	partial	SF	slip potential
(111) ABC	AB: $\frac{1}{2}[0\bar{1}1]$	0.328	δA: $\frac{1}{6}[11\bar{2}]$	−0.227	×
	AC: $\frac{1}{2}[\bar{1}01]$	0.064	δB: $\frac{1}{6}[1\bar{2}1]$	0.342	
	BC: $\frac{1}{2}[\bar{1}10]$	−0.263	δC: $\frac{1}{6}[\bar{2}11]$	−0.115	×
(1$\bar{1}\bar{1}$) ABD	AB: $\frac{1}{2}[0\bar{1}1]$	0.143	Aγ: $\frac{1}{6}[\bar{1}\bar{2}1]$	0.087	×
	DA: $\frac{1}{2}[110]$	−0.007	Bγ: $\frac{1}{6}[\bar{1}1\bar{2}]$	−0.161	
	DB: $\frac{1}{2}[101]$	0.136	Dγ: $\frac{1}{6}[211]$	0.074	×
**observed** **($\bar{1}1\bar{1}$)** **ACD**	AC: $\frac{1}{2}[\bar{1}01]$	−0.170	Aβ: $\frac{1}{6}[\bar{2}\bar{1}1]$	**−0.123**	
	DC: $\frac{1}{2}[011]$	−0.128	Cβ: $\frac{1}{6}[1\bar{1}\bar{2}]$	0.172	×
	DA: $\frac{1}{2}[110]$	0.042	Dβ: $\frac{1}{6}[121]$	**−0.049**	
($\bar{1}\bar{1}$1) BCD	BC: $\frac{1}{2}[\bar{1}10]$	−0.313	Bα: $\frac{1}{6}[\bar{2}1\bar{1}]$	−0.395	
	DB: $\frac{1}{2}[101]$	0.371	Cα: $\frac{1}{6}[1\bar{2}\bar{1}]$	0.147	×
	DC: $\frac{1}{2}[011]$	0.058	Dα: $\frac{1}{6}[112]$	0.247	×

Grain II					
plane	perfect	SF	partial	SF	slip potential
(511) ABC′	AB: $\frac{1}{2}[0\bar{1}1]$	0.423	δ'A: $\frac{1}{18}[\bar{1}7\bar{2}]$	−0.367	×
	AC′: $\frac{1}{6}[14\bar{1}]$	0.213	δ'B: $\frac{1}{18}[\bar{1}\bar{2}7]$	0.366	
	BC′: $\frac{1}{6}[1\bar{1}\bar{4}]$	−0.211	δ'C′: $\frac{1}{18}[2\bar{5}\bar{5}]$	0.001	
($\bar{1}$11) ABD	AB: $\frac{1}{2}[0\bar{1}1]$	−0.143	Aγ: $\frac{1}{6}[\bar{1}\bar{2}1]$	−0.087	
	DA: $\frac{1}{2}[110]$	0.007	Bγ: $\frac{1}{6}[\bar{1}1\bar{2}]$	0.161	×
	DB: $\frac{1}{2}[101]$	−0.136	Dγ: $\frac{1}{6}[211]$	−0.074	
**observed** **($\bar{1}1\bar{5}$)** **AC′D**	AC′: $\frac{1}{6}[1\bar{4}\bar{1}]$	−0.384	Aβ': $\frac{1}{18}[\bar{2}\bar{7}\bar{1}]$	**−0.244**	
	DC′: $\frac{1}{6}[4\bar{1}\bar{1}]$	−0.347	C′β': $\frac{1}{18}[\bar{5}52]$	0.422	×
	DA: $\frac{1}{2}[110]$	0.038	Dβ': $\frac{1}{18}[72\bar{1}]$	**−0.178**	
($\bar{1}\bar{5}$1) BC′D	BC′: $\frac{1}{6}[1\bar{1}\bar{4}]$	−0.242	Bα': $\frac{1}{18}[\bar{2}\bar{1}\bar{7}]$	−0.406	
	DB: $\frac{1}{2}[101]$	0.461	C′α': $\frac{1}{18}[\bar{5}25]$	0.013	×
	DC′: $\frac{1}{6}[4\bar{1}\bar{1}]$	0.220	Dα': $\frac{1}{18}[7\bar{1}2]$	0.393	×

Fig. S3. An atomic structure model built to reproduce the geometrical relationship between Grains-I and -II in Fig. 4(a). The Schmid factor for each of possible slip systems are summarized in the attached tables. The notifications of AB and δA mean the slip direction from A to B and δ to A, respectively, and the sign of Schmid factor corresponds to the defined slip direction. In the case of partial dislocation slips, only in either the positive or negative sliding direction can be activated due to the geometrical limitation of the crystal structure. The non-activated slip direction is checked in the column named slip potential. The Schmid factor for the observed stacking fault in the Grain-II is calculated to be 0.244 on plane AC'D. The Schmid factor for the observed stacking fault nucleated from the Σ3 boundary to the grain-I on plane ACD is relatively low, 0.12 and 0.049. The possible direction of the Burgers vector nucleated from the Σ3 boundary to the Grain-I under the uniaxial stress state is almost opposite direction of the Burgers vector approaching to the Σ3 boundary in the Grain-II, indicating the stacking fault nucleation in Grain-I requires the additional local stress concentration, which can realize the nucleation of a reasonable partial dislocation, as shown in a gray colored dislocation in the broken circle, which does not leave the large residual Burgers vector at the Σ3 boundary.
